# A Mathematical Analysis of a Biomechanical Model for an Innovative Spinal Decompression and Correction System for the Conservative Treatment of Scoliosis

**DOI:** 10.3390/bioengineering12050509

**Published:** 2025-05-11

**Authors:** Yi Jie, Mingwen Zhang, Mengyao Li, Changliang Luo, Anqin Dong, Yu-Yan Luo, Pengyuan Zheng, Xinmin Zhang, Zhihua Liu, Jing Li, Man-Sang Wong, Annie Yan Wang, Christina Zong-Hao Ma, Ming Zhang

**Affiliations:** 1 Department of Rehabilitation Medical Engineering, The Fifth Affiliated Hospital, Zhengzhou University, Zhengzhou 450052, China; yi620.jie@connect.polyu.hk (Y.J.); mengyao0930@outlook.com (M.L.); anqindong@163.com (A.D.); medp7123@126.com (P.Z.); jingliljlj@163.com (J.L.); 2 Department of Biomedical Engineering, The Hong Kong Polytechnic University, Hong Kong SAR, China; chang-liang.luo@connect.polyu.hk (C.L.); yuyan-laura.luo@connect.polyu.hk (Y.-Y.L.); m.s.wong@polyu.edu.hk (M.-S.W.); ming.zhang@polyu.edu.hk (M.Z.); 3 Research Institute of Rehabilitation Medicine, Henan Academy of Innovations in Medical Science, Zhengzhou 451163, China; 17737316777@163.com; 4 School of Mechanical and Power Engineering, Zhengzhou University, Zhengzhou 450001, China; zmw77zq@163.com (M.Z.); liuzhihua@zzu.edu.cn (Z.L.); 5 Research Institute for Sports Science and Technology, The Hong Kong Polytechnic University, Hong Kong SAR, China; 6 Research Institute for Smart Ageing, The Hong Kong Polytechnic University, Hong Kong SAR, China

**Keywords:** scoliosis, correction system, mathematical model, calculation, biomechanics

## Abstract

Scoliosis is a three-dimensional deformity of the spine that can lead to a series of physical and psychological problems. Appropriate controlling forces should be applied to prevent the curve’s progression and even correct the deformity. The aims of this study were to develop a biomechanical model that can quickly estimate the optimal positions and magnitudes of the controlling forces for treating scoliosis and to analyze the interaction between longitudinal traction and lateral forces. Based on the scoliotic curve information that was extracted and simulated from the computed tomography data of patients, a mathematical model of scoliosis was established via the Timoshenko beam theory. The model could be optimized to provide precise and effective treatment for patients with different scoliosis curve patterns. The relationship between the corrective force position, magnitude, and the treatment effect on scoliosis could be obtained using this model. This study provides a biomechanical theoretical basis for determining the magnitude, position, and sequence of applying controlling forces on spines for patients with scoliosis.

## 1. Introduction

Scoliosis is a three-dimensional (3D) deformity of the spine characterized by abnormalities in the sagittal, coronal, and transverse planes [1]. It is defined as a deviation of the spine to the left or right side from the central axis of the sacrum, with a Cobb angle greater than 10° in the coronal plane, and it is accompanied by a certain degree of rotation on standing radiographs [1]. Scoliosis is currently one of the top three conditions endangering the health of adolescents [1]. Changes in the morphology of the spine can lead to a series of problems, such as abnormal posture, the degeneration of cardiopulmonary function, and decreased muscle strength and endurance. Such problems can seriously affect the physical and mental health of adolescents [2].

The treatment of scoliosis is typically divided into surgical and non-surgical methods. Clinicians often refer to the foundational work of White and Panjabi on the clinical biomechanics of scoliosis [3] to determine the application of the axial and transverse forces during treatment. The widely accepted threshold for surgery is a Cobb angle of 45° or greater [4]. However, due to the significant risks associated with surgery, it is not often chosen; thus, non-surgical methods are typically employed in most cases [5]. Common non-surgical treatments involve traction, orthotic treatment, and Physiotherapeutic Scoliosis Specific Exercises (PSSEs) [6]. Both surgical and non-surgical methods apply corrective forces to scoliotic curves to gradually shift them toward the midline. It is of great significance to explore the biomechanical principle of scoliosis correction.

Biomechanics, a cross-disciplinary field combining both mechanics and biology, aims to address problems that cannot be effectively solved within the realm of medicine alone, such as the correction of scoliosis. The biomechanical analysis of scoliosis allows for precise control of the manner and magnitude of applied corrective loads. Smith et al. introduced several biomechanical parameters, such as structural bending stiffness, axial stiffness, and range of motion, under different loading modules to describe the functional biomechanics of the spine [7]. Orne et al. designed a two-dimensional discrete parameter model which considers the human body as a system of springs, dampers, and rigid bodies and derived the axial force equation for intervertebral disks [8]. Iorio et al. conducted a biomechanical analysis of degenerative spinal diseases and discovered that as the height of the intervertebral disk decreased, the range of axial rotation of a spinal unit increased, which placed increased mechanical load on the posterior facet joints [9]. Changes in the stress–strain relationship of spinal disks were also reported [9]. Although previous studies have conducted biomechanical analyses of the spine, there is a lack of studies focusing on mathematical modeling and analysis for scoliosis, especially those that integrate the functions and characteristics of some corrective devices to make correction models more practically valuable.

Both traditional traction techniques and the use of custom-made scoliosis braces for scoliosis correction involve the application of forces in a single direction only [10]. Traction therapy involves pulling at both ends of the scoliotic curve longitudinally to ‘pull’ the spine back to its normal position through axial loading [11]. In contrast, custom-made scoliosis braces utilize the ‘three-point pressure’ principle and apply transverse corrective forces to the apical vertebra and the two upper- and lower-end vertebrae of the scoliotic curve to ‘push’ the spine back into its normal position through lateral forces [11]. However, to the best of the authors’ knowledge, so far, no previous studies have combined longitudinal decompression and transverse correction together when developing a biomechanical model for scoliosis correction.

Recently, a novel digitalized/mechanical 3D spinal decompression and correction system and treatment protocol were preliminarily evaluated through three clinical studies. Specifically, a single-center randomized controlled trial (RCT) involving 110 patients with adolescent idiopathic scoliosis (AIS) reported a significant in-brace correction ratio of 0.85 ± 0.11 (thoracic/thoracolumbar) and a significant 45.4% improvement in comfort after 2–3 days of the digitalized scoliosis correction treatment [12]. A case–controlled clinical study on 40 AIS patients showed that a single 30 min digitalized scoliosis correction session significantly improved spinal rotation by 7.8–8.2% and body height by 1.0 cm, outperforming traditional Schroth exercises [13]. A more recent feasibility study involving 19 patients with mild, moderate, and severe AIS observed that a 28 min session led to a 40.1% immediate reduction in the Cobb angle, along with a decrease in the required corrective force over seven decompression–correction cycles [14]. Although these three previous clinical studies confirmed the short-term efficacy of the newly developed device and treatment protocol, the underlying biomechanical and mathematical mechanisms remain unclear. Moreover, the application of decompressive and corrective forces in these studies was based on prior research related to low back pain and bracing treatments without any mathematical or biomechanical validation. Therefore, it is necessary to further develop a biomechanical model to understand how different forces affect spinal curvature and quantify these effects using mathematical methods.

Therefore, this study aimed to (1) develop a mathematical and biomechanical model of a novel spinal decompression and correction device for the conservative treatment of scoliosis through differential equations and (2) analyze the mathematical and biomechanical principles of scoliosis correction, investigating the relationships among the longitudinal force, lateral force, force positions, and the resulting Cobb angle. It is expected that the findings of this study could provide a theoretical basis for the conservative management of scoliosis for future research and clinical practice.

## 2. Biomechanical Model for Analysis of Scoliosis Correction System

### 2.1. The Principles of the Scoliosis Correction System

**Figure 1:**
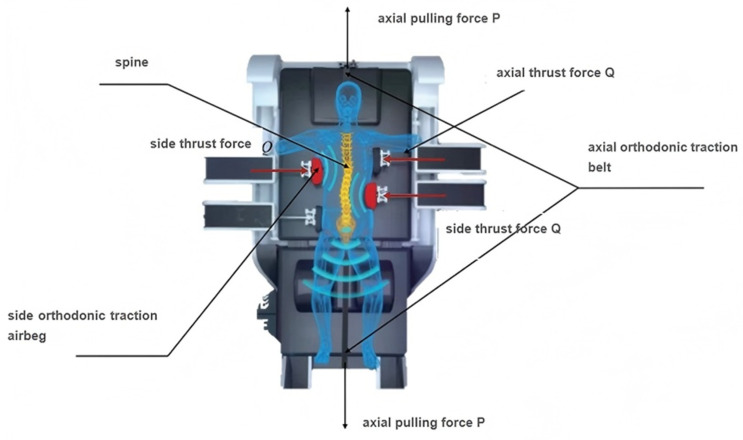
Schematic diagram of scoliosis correction device.

A schematic diagram of the scoliosis correction device that was analyzed in this study is shown in Figure 1. This device provided the digitalized 3D spinal decompression and correction treatment for the abnormal 3D structure of a scoliotic spine by combining both lateral and axial loads. In this context, the term ‘decompression’ refers to the application of traction forces along the longitudinal axis of the spine (Z-axis) through traction belts and the control system of the device. The decompression function aimed to reduce the axial pressure on spine while the patient was in a supine position. In essence, it combined traction force with the ‘three-point pressure’ principle to treat scoliosis.

Axial loads were applied through traction belts that were fixed to the headrest and control cabinet. The bed body, which was controlled using a mechanical power system, could regulate the magnitude of the axial spinal decompressive load separately. The lateral corrective force came from the bilateral correction devices and intelligent airbags at the sides of the bed body. The bilateral correction devices and the airbags could move upward and downward along the bed body to achieve the provision of precise pressure on the spine. The magnitudes of both the axial and lateral loads, which were measured using sensors and displayed on the control screen of the system, could be automatically adjusted based on the patient’s actual response during spinal decompression and correction.

In this study, a biomechanical model was proposed and developed to incorporate the features of various scoliosis correction modalities. The model was used to theoretically analyze and understand the treatment process. Specifically, it examined the magnitude, angle, and method of force application on an AIS participant.

### 2.2. Materials and Methods

**Figure 2:**
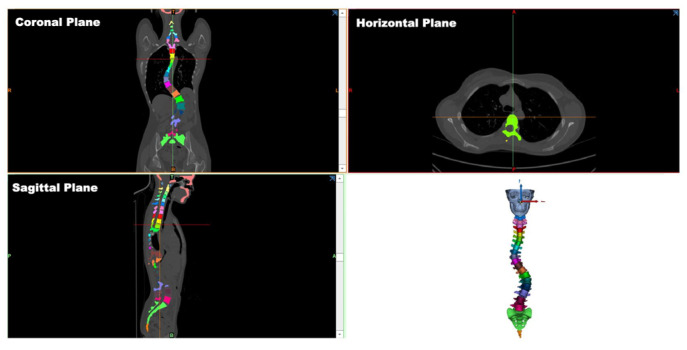
Orthographic views of each segment of the spine on CT images.

**Figure 3:**
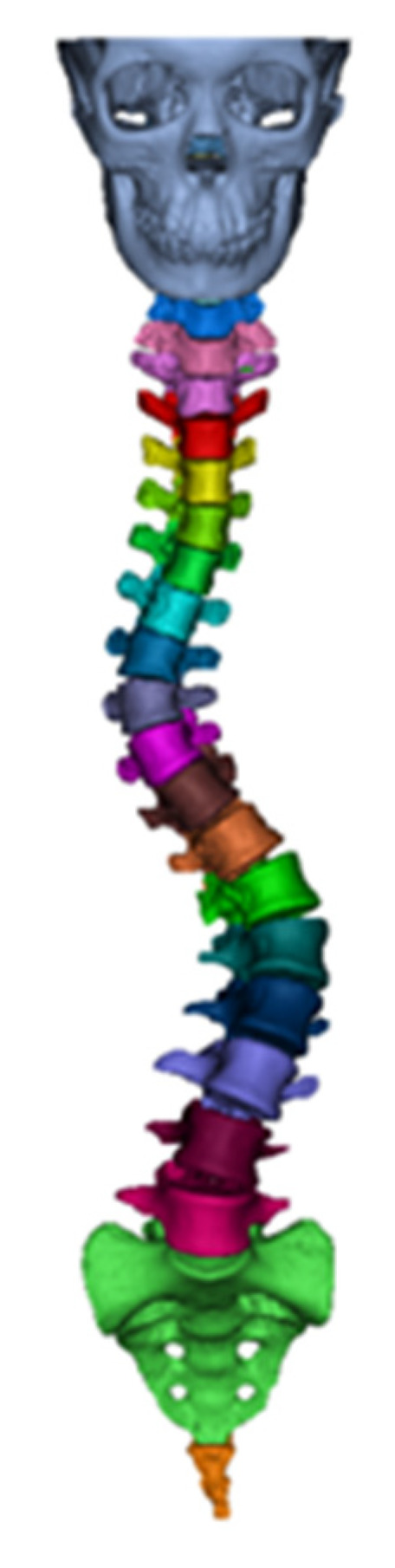
Illustration of separated full spine surface mesh model.

To investigate the biomechanical effects of the 3D spinal decompression and correction device on scoliosis, a 3D finite element model of the scoliotic spine was developed. This model was designed to support the collection and analysis of spinal coordinate data, which are critical for understanding the biomechanical parameters and curvature characteristics of a scoliotic spine. A 64-slice spiral computed tomography (CT) scan was performed on a volunteer with scoliosis to obtain 723 two-dimensional (2D) tomographic CT images. The scanning data were directly saved in DICOM format. The Mimics 21.0 software (Materialise, Leuven, Belgium) was used to open and incorporate the spinal CT image data in DICOM format (Figure 2), with the threshold range set as 226–2976 HU. Upon utilizing the processing functions of the software, the connecting parts of each vertebral segment were manually separated, with the cavities filled. This process generated a 3D model of the skull and the entire spine, which was then optimized to obtain a separated full-surface mesh model of the entire spine (Figure 3).

To determine the maximum deviation of a scoliotic spine and calculate the biomechanical parameters, the characteristic curve of a patient’s scoliotic curvature was extracted. This process required establishing a mathematical model based on the finite element scoliotic model. Firstly, a rectangular coordinate system was established in the coronal plane. The intersection of the lower edge of the most inferior vertebra of the scoliotic curvature and the midline of the spine served as the origin. The positive direction of the x-axis was defined as vertically upward along the midline of the sacrum. Within this established coordinate system, the Mimics software was used to collect the coordinates of the position points along the midline of the spine in the patient’s CT scan at an equal spacing of 7 mm. As shown in Table 1, a total of 29 sets of spinal coordinate data were collected from the finite element model. These points, which ranged from the lower edge of L5 to the upper end of the T10 vertebra, formed the basis for analyzing the biomechanical effects and curvature deviations of the spine during treatment.

### 2.3. An Analysis of the Biomechanical Model of the Scoliosis Decompression and Correction Device

**Figure 4:**
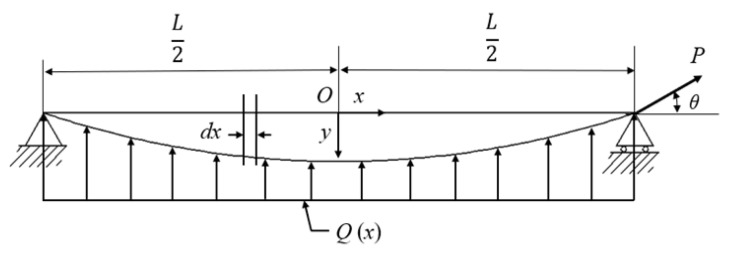
Schematic diagram of biomechanical model for spinal decompression and correction device.

The curve of the scoliotic curvature of the patient’s spine was mechanically simplified as a bent Timoshenko beam-column [15,16]. As shown in Figure 4, the displacement of the scoliotic curvature to the midline corresponds to the bending displacement of the beam-column. In this figure, *Q*(*x*) represents a uniformly distributed lateral corrective load perpendicular to the lateral surface of the spine, *P* represents the axial decompressive load along the length of the spine, *x* indicates the measured position of the spine, *y* indicates the lateral displacement of the bent beam-column, and the origin, *O*, is located at the midpoint of the horizontal position of the spine.

Therefore, the total lateral displacement of the beam-column is expressed as the sum of the initial lateral displacement of the bent beam-column and the lateral displacement caused by the combined action of axial and lateral loads as follows:

(1) $$y(x)=y_{0}(x)+y_{1}(x)$$

The initial lateral displacement of the bent beam-column was determined through curve fitting. Based on the 29 sets of spinal coordinate point data obtained from the patient (Table 1), and with the marked point *O* as the origin of the curve (Figure 4), curve fitting was performed using the nonlinear curve fitting function of the Origin 2018 software (OriginLab, Northampton, MA, USA) to obtain the initial lateral displacement curve as follows:

(2) $$y_{0}(x)=-33.86561x^{3}-3.55545x^{2}+0.3189x+0.03345$$

According to the Timoshenko beam theory [17], the functional relationship between the initial lateral displacement and the lateral displacement caused by the combined action of the axial and lateral loads is as follows:

(3) $$EI\frac{d^{4}y_{1}}{dx^{4}}+P\frac{d^{2}y_{1}}{dx^{2}}=Q(x)-P\frac{d^{2}y_{0}}{dx^{2}},\;x\in \left[-\frac{L}{2},\frac{L}{2}\right]$$

where *EI* represents the bending stiffness of the human spine, and the boundary conditions are as follows:

(4) $$y_{1}(-\frac{L}{2})=y_{1}(\frac{L}{2})=0$$

(5) $$EI\frac{d^{2}y_{1}}{dx^{2}}\left(-\frac{L}{2}\right)=k_{s}\frac{d^{2}y_{1}}{dx^{2}}\left(-\frac{L}{2}\right)$$

(6) $$-EI\frac{d^{2}y_{1}}{dx^{2}}\left(\frac{L}{2}\right)=k_{i}\frac{d^{2}y_{1}}{dx^{2}}\left(\frac{L}{2}\right)$$

$k_{i}$ is expressed as

(7) $$k_{i}=\frac{3EI}{L_{i}}$$

where $L_{i}$ represents the length of the lower part of the spine that is not included in the scoliotic curve. The proportional constants $k_{s}$ and $k_{i}$ are elastic constants [16]. During the treatment, the patient laid on the spinal decompression and correction device in a fixed position, with lateral and axial loads applied to correct the lateral spinal deviation. During this process, the body only received support at specific positions, and the neck was not constrained, allowing for limited head movement. Therefore, no rotational support was applied in this model, and $k_{s}$ was set as zero [16].

The values of the proportional constants, $k_{s}$ and $k_{i}$, could be adjusted according to the scoliotic condition of the spine during the specific decompression and correction treatment process and the adjustment of the device based on the location of the scoliotic curve. Therefore, the values of $k_{s}$ and $k_{i}$ varied for patients with different types of scoliosis and based on various adjustments of the decompression and correction device setup and different restraining conditions. In this case, a set of values for $k_{s}$ and $k_{i}$ were determined to customize the model for individual patients. Based on these settings, the spinal decompression and correction device was used to provide personalized treatment plans to improve patient outcomes.

To explore the interaction between the axial and lateral loads in the correction of scoliosis, and to determine the specific magnitudes of the loads and the treatment period required for scoliosis correction, detailed modeling of the scoliotic curvature of the patient was conducted, and the detailed parameters are illustrated in Table 2.

The length of the curve was determined from the data shown in Table 2. The bending stiffness value refers to the average value obtained from the experimental research results of a previous study [18]. The axial decompressive load typically ranges between 30% and 50% of a patient’s body weight, as suggested by prior studies [19]. In this study, we selected 40% of the patient’s body weight as the axial decompressive load for each treatment session based on the commonly recommended range and the specific requirements of the experimental setup [15]. Regarding transverse corrective loads, prior studies showed that applying 20 N, 40 N, or 60 N led to improved correction effects, with greater forces typically yielding better results [20,21]. Additionally, Li et al. applied transverse corrective loads of 10%, 15%, 20%, 25%, and 30% of the patient’s body weight and found that a transverse corrective load of 25% of the patient’s body weight produced a better correction effect for mild to moderate and severe scoliosis than other loads [22]. Based on these findings, the current study adopted 25% of the patient’s body weight as the transverse corrective load.

As shown in Figure 4, the axial load angle refers to the angle between the axial load and the beam-column. In the scoliosis decompression and correction device, the axial decompressive load could be applied within an angle range of 0° to 90°. For the following analysis, the mathematical models are represented by differential equations, where the initial application of the axial decompressive load is assumed to act strictly along the axial plane of the spine. Under this setup, α = 0°.

#### 2.3.1. Treating Scoliosis with Only the Axial Decompressive Load

In this case, the transverse load was set to zero. Upon taking into account of all the parameters, Equation (3) became

(8) $$15.1\frac{d^{4}y_{1}}{dx^{4}}-P\cdot \cos\left(\alpha \right)\cdot \frac{d^{2}y_{1}}{dx^{2}}=P\cdot \cos\left(\alpha \right)\cdot \frac{d^{2}y_{0}}{dx^{2}}$$

At this point, the boundary conditions changed to

(9) $$y_{1}(-0.098)=y_{1}(0.098)=0$$

(10) $$15.1\frac{d^{2}y_{1}}{dx^{2}}\left(-0.098\right)=0$$

(11) $$-15.1\frac{d^{2}y_{1}}{dx^{2}}\left(0.098\right)=0$$

Upon taking into account of the above equations, the total lateral displacement was determined as follows:

(12) $$\begin{array}{l} y(x)=\frac{15.1}{P}\cdot \frac{19.91297868(e^{0.196\cdot \sqrt{\frac{P}{15.1}}}+1)-7.1109(e^{0.196\cdot \sqrt{\frac{P}{15.1}}}-1)}{e^{0.294\cdot \sqrt{\frac{P}{15.1}}}-e^{-0.098\cdot \sqrt{\frac{P}{15.1}}}}\cdot e^{-\sqrt{\frac{P}{15.1}}\cdot x}\\ +\frac{15.1}{P}\cdot \frac{-19.91297868(e^{0.196\cdot \sqrt{\frac{P}{15.1}}}+1)-7.1109(e^{0.196\cdot \sqrt{\frac{P}{15.1}}}-1)}{e^{0.294\cdot \sqrt{\frac{P}{15.1}}}-e^{-0.098\cdot \sqrt{\frac{P}{15.1}}}}\cdot e^{\sqrt{\frac{P}{15.1}}\cdot x}\\ -0.00634530612x+\frac{3068.224266x}{P}-0.0006965418+\frac{107.37459}{P} \end{array}$$

**Figure 5:**
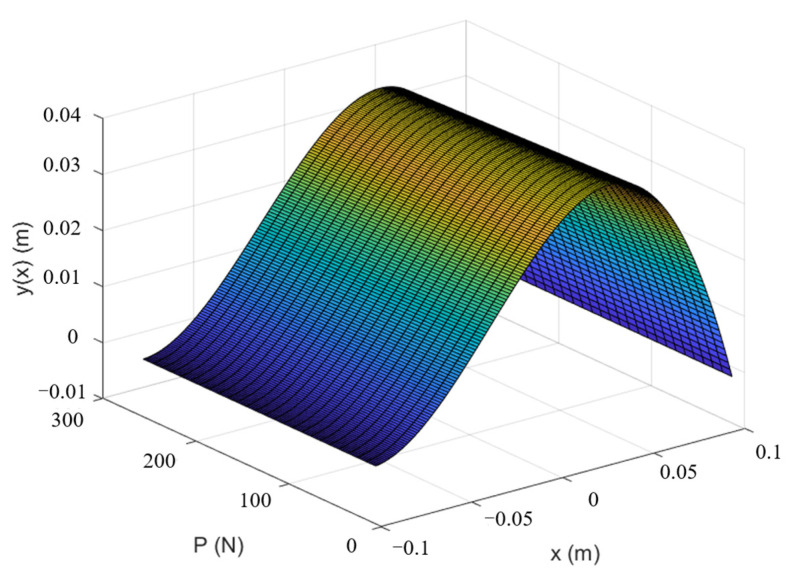
Schematic diagram of total lateral displacement under axial decompressive load only.

From Equation (12), it can be observed that the total lateral displacement y(x) depends on the position of the spine and the magnitude of the axial decompressive load. The interaction between them is illustrated in Figure 5, where $x\in \left[-\frac{L}{2},\frac{L}{2}\right]$ and $P\in \left[0,260\right]$.

**Figure 6:**
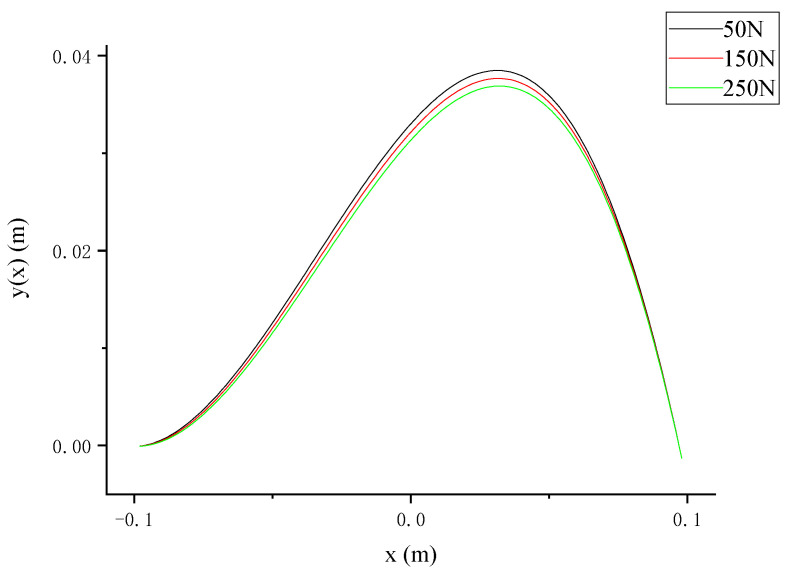
Schematic diagram of total lateral displacement under different axial decompressive loads.

As shown in Figure 5, the total lateral displacement did not exhibit significant changes across the entire range of axial decompressive loads ranging from 0 to 260 N. To more clearly illustrate the impact of specific axial decompressive loads on the total lateral displacement, this study selected three representative values of 50 N, 150 N, and 250 N and extracted their respective total lateral displacement curves. These lateral displacement curves were used to generate a schematic diagram highlighting the effect of different axial decompressive loads on the total lateral displacement (Figure 6).

As shown in Figure 6, the total lateral displacement gradually decreased with the increasing axial decompressive load. This indicates that the higher the applied axial load, the closer the scoliotic curve approaches zero, suggesting a better correction effect. Through an analysis of Equation (11) and Figure 5, it was determined that when *x* = 0.095, the total lateral displacement y(x) approached zero, indicating successful scoliosis correction. Upon substituting the value of *x* back into Equation (12), the total lateral displacement y(x) represented solely by the axial decompressive load *P* was obtained as follows:

(13) $$\begin{array}{l} y(P)=\frac{15.1}{P}\cdot \frac{19.91297868(e^{0.196\cdot \sqrt{\frac{P}{15.1}}}+1)-7.1109(e^{0.196\cdot \sqrt{\frac{P}{15.1}}}-1)}{e^{0.294\cdot \sqrt{\frac{P}{15.1}}}-e^{-0.098\cdot \sqrt{\frac{P}{15.1}}}}\cdot e^{-\sqrt{\frac{P}{15.1}}\cdot 0.095}\\ +\frac{15.1}{P}\cdot \frac{-19.91297868(e^{0.196\cdot \sqrt{\frac{P}{15.1}}}+1)-7.1109(e^{0.196\cdot \sqrt{\frac{P}{15.1}}}-1)}{e^{0.294\cdot \sqrt{\frac{P}{15.1}}}-e^{-0.098\cdot \sqrt{\frac{P}{15.1}}}}\cdot e^{\sqrt{\frac{P}{15.1}}\cdot 0.095}\\ -0.0012993458814+\frac{398.85589527}{P} \end{array}$$

For Equation (13), upon setting the total lateral displacement of the spine to zero, the total axial decompressive load was calculated as follows:

P=35351N

As these loads could not be applied to the scoliotic spine all at once, the recommended maximum loads were used per session. The application of both axial and transverse loads is commonly based on the patient’s maximum tolerance level [23] or a percentage of their body weight [24]. However, no unified standard exists, and the therapeutic effect remains uncertain. Ernest et al. selected 33% to 50% of the patient’s body weight as the maximum decompression/traction force based on the patient’s tolerance [25]. They found that after a period of traction, the patients’ scoliotic curvature improved significantly. Jacques et al. treated patients by gradually increasing the traction force according to the patient’s body shape while setting the maximum force as reaching only 30% to 50% of the patient’s body weight [26]. Since the maximum dose per session is a proportion of the patient’s body weight, the correction device needs to apply the force over multiple sessions until the cumulative total force reaches the calculated target.

**Figure 7:**
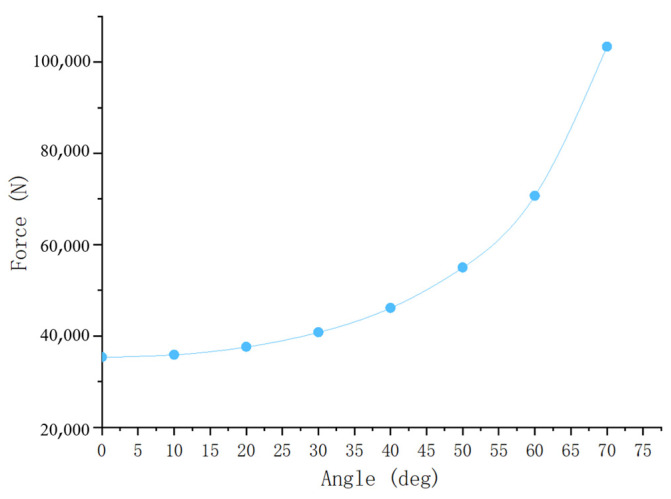
Schematic diagram of relationship between total axial decompressive load and traction angle.

The axial decompressive load calculated in this study was based on the condition of α = 0°, but the correction device can apply decompression/traction at different angles. Moreover, the device allows patients to adjust the angle when they feel discomfort at a certain angle. However, it should be noted that changes in the angle had a practical impact on the total axial decompressive load. Thus, the current study also considers the influence of the traction angle on spinal curvature correction (Figure 7).

As shown in Figure 7, the total axial decompressive load increased nonlinearly with the traction angle, highlighting the importance of selecting an appropriate angle. In this case, the total axial load required to eliminate the scoliotic curve was calculated as 35,351 N. As each correction session was limited to a percentage of body weight, the patient was required to undergo multiple sessions until the cumulative force reached this value. This result also enabled an estimation of the treatment cycle and planning of the sessions.

Additionally, based on the spinal position coordinate data shown in Table 1, the patient’s maximum scoliotic deviation was measured as 40.04 mm. Two additional sets of spinal data with maximum scoliotic deviations of 32.07 mm and 26.90 mm were also analyzed. Upon using the same method described above, we calculated that the corresponding total axial decompressive loads shall be 25,852 N and 21,370 N, respectively. A comparison revealed that patients with mild to severe scoliosis required a smaller total axial decompressive load and had a shorter treatment cycle than those with moderate to severe scoliosis.

The above analysis was conducted under the assumption of treating scoliosis solely with the axial decompressive load. Similarly, the analysis of treating scoliosis solely with the transverse corrective load would yield similar results, where a greater transverse corrective load would result in a scoliotic curve closer to zero and better correction effects. Moreover, the more severe the scoliosis, the greater the transverse corrective load required. Therefore, further elaboration is not necessary here. In the following section, we analyze the combined effect of the axial decompressive and transverse corrective loads on the treatment of scoliosis.

#### 2.3.2. The Combined Effect of Axial Decompressive and Lateral Corrective Loads for Treating Patients with Scoliosis

For this analysis, upon considering all specific parameters, Equation (14) is as follows:

(14) $$15.1\frac{d^{4}y_{1}}{dx^{4}}-P\frac{d^{2}y_{1}}{dx^{2}}=-Q+P\frac{d^{2}y_{0}}{dx^{2}}$$

The boundary conditions remain the same as those in Equations (9)–(11), so the total lateral displacement can be determined as

(15) $$\begin{array}{l} y(x)=\frac{Q}{2P}\cdot x^{2}+\frac{15.1}{P}\cdot \frac{19.91297868(e^{0.196\cdot \sqrt{\frac{P}{15.1}}}+1)P-(Q+7.1109P)(e^{0.196\cdot \sqrt{\frac{P}{15.1}}}-1)}{(e^{0.294\cdot \sqrt{\frac{P}{15.1}}}-e^{-0.098\cdot \sqrt{\frac{P}{15.1}}})P}\cdot e^{-\sqrt{\frac{P}{15.1}}\cdot x}\\ +\frac{15.1}{P}\cdot \frac{-19.91297868(e^{0.196\cdot \sqrt{\frac{P}{15.1}}}+1)-(Q+7.1109P)(e^{0.196\cdot \sqrt{\frac{P}{15.1}}}-1)}{(e^{0.294\cdot \sqrt{\frac{P}{15.1}}}-e^{-0.098\cdot \sqrt{\frac{P}{15.1}}})P}\cdot e^{\sqrt{\frac{P}{15.1}}\cdot x}\\ -\frac{0.004802Q}{P}-0.00634530612x+\frac{3068.224266x}{P}-0.0006965418+\frac{15.1(Q+7.1109P)}{P} \end{array}$$

**Figure 8:**
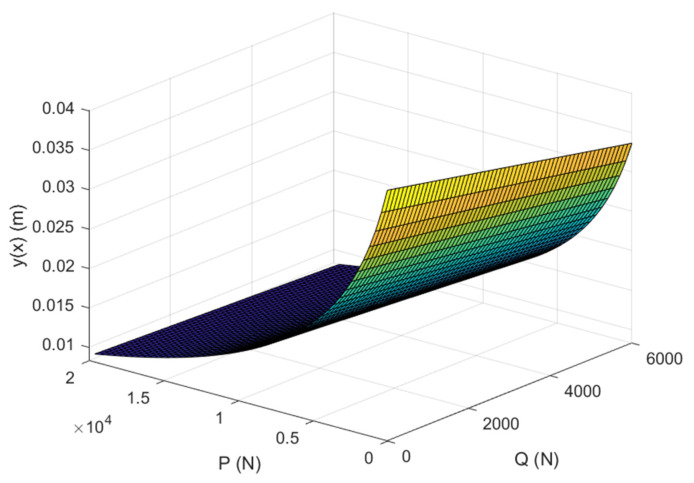
Schematic diagram of total lateral displacement of spine under combined action of axial decompressive and lateral corrective loads.

Due to the multiple unknown numbers in the above equation, it was difficult to determine the relationship between the axial decompressive load and the lateral corrective load in this model. Therefore, this study discussed the maximum displacement of the patient’s scoliosis, which was shown as the 20th data point in Table 1, where *x* = 0.035 and the maximum displacement of scoliosis was 40.04 mm. The values were substituted back into the curve. The MATLAB R2023b software (MathWorks, Natick, MA, USA) was used to obtain the total lateral displacement of the spine under the combined action of the axial decompressive and lateral corrective loads, as shown in Figure 8.

As shown in Figure 8, with the increase in the axial decompressive load or lateral corrective load, the total lateral displacement of the spine gradually decreased. When compared with the case of applying the axial decompressive load only, the total lateral displacement decreased with the increase in the lateral corrective load, while the axial decompressive load remained unchanged. Therefore, the combination of traction and the ‘three-point pressure’ principle had a positive impact on the treatment of scoliosis. For the same total lateral displacement, a higher lateral corrective load resulted in a lower required axial decompressive load. This reduced the cumulative treatment dose and shortened the treatment cycle, thereby improving efficiency.

#### 2.3.3. Interaction Between Axial Decompressive and Lateral Corrective Loads

**Figure 9:**
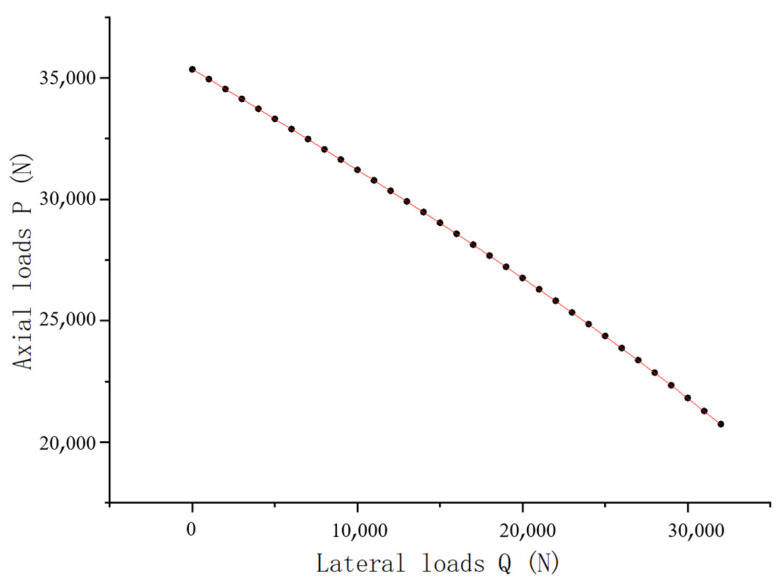
Schematic diagram of interaction between axial and lateral loads.

The analysis of Figure 5 and Equations (8)–(13) revealed that when *x* = 0.095, the total lateral displacement y(x) approached zero, indicating successful scoliosis correction. At this point, the lateral load gradually increased from zero, and a series of values for the total axial decompressive load under different magnitudes of lateral corrective load were obtained (Figure 9).

As shown in Figure 9, the value of the total axial decompressive load gradually decreased as the lateral corrective load increased. Although the relationship between the axial decompressive and lateral corrective loads appeared to be linear in Figure 9, the decrease in the total axial decompressive load led to a faster increase in the lateral corrective load. However, the numerical difference was relatively small, making it not prominent in Figure 9.

The interaction described above was based solely on an analysis of the AIS volunteer in this study. It was not tested on other patients. Therefore, the following analysis further explored the interaction when the effect of the axial decompressive load was greater than that of the lateral corrective load, and when the effect of the lateral corrective load was greater than that of the axial decompressive load during the combined treatment. This allowed us to determine the appropriate application sequence and dominant load, leading to personalized and more efficient treatment.

**Figure 10:**
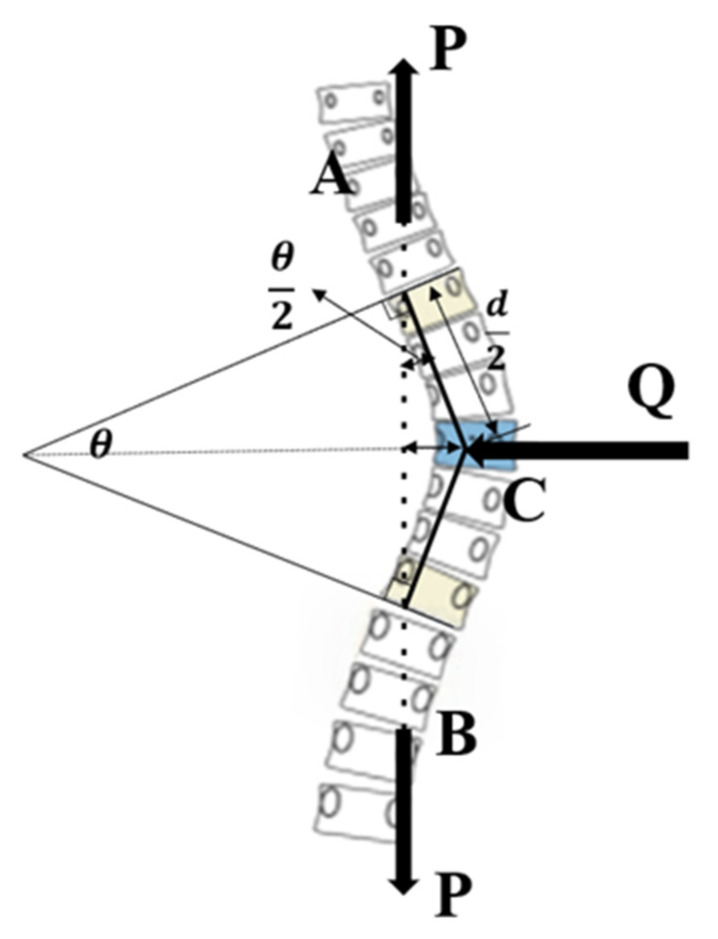
A schematic diagram of the Cobb angle and applied forces in a patient with scoliosis. Yellow indicates the upper and lower end vertebrae, and blue indicates the apical vertebra.

The Cobb angle, a standard index of scoliosis severity, was measured on full-spine anteroposterior radiographs. A larger angle generally indicates more severe scoliosis. It is defined as the angle between lines drawn along the upper and lower endplates of the most tilted vertebrae [27], as indicated by *θ* in Figure 10. In the figure, the lateral corrective load is applied at the apex of the curve and labeled as C, while the axial decompressive loads are applied at points A and B at both sides. The relative corrective abilities of these two loads could be derived from the magnitude of the bending moment at apex C, as explained in the following analysis.

The bending moment at apex C of the scoliosis curve when the spine is subjected to an axial decompressive load is given by the following expression:

(16) $$M_{P}=P\cdot \frac{d}{2}\cdot \sin\frac{\theta }{2}$$

From this bending moment expression, it can be observed that a greater Cobb angle indicates more severe scoliosis and a greater bending moment at apex C. This also suggests that the axial decompressive load has better corrective ability.

When the spine was subjected to a lateral corrective load, according to the principle of interaction, endpoints A and B experienced a reactive force equivalent to half of the lateral decompressive load. At this time, the force at endpoint A generated a bending moment at apex C of the scoliotic curve, shown as follows:

(17) $$M_{Q}=\frac{Q}{2}\cdot \frac{d}{2}\cdot \cos\frac{\theta }{2}$$

Similarly to endpoint B, where the effect of the lateral corrective load was the opposite of that of endpoint A, the corrective ability of the lateral corrective load gradually decreased as the Cobb angle increased.

**Figure 11:**
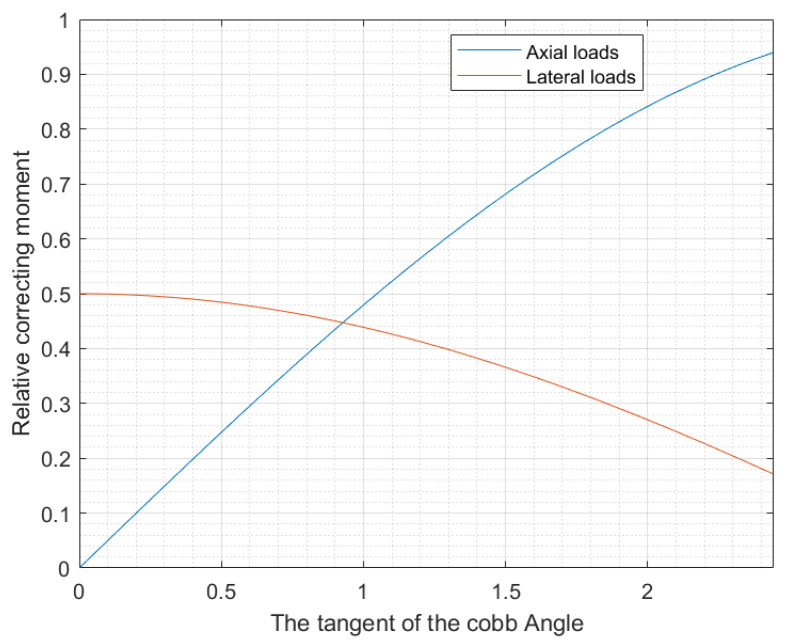
Schematic diagram of relationship between relative correction moment of loads and Cobb angle.

From the above analysis, it was concluded that the combination of the axial decompressive and lateral corrective loads corrected the scoliosis curvature with less total force, reducing the treatment cycle for patients and enabling them to recover faster from the disease. The MATLAB R2023b software (MathWorks, Natick, MA, USA) was used to further explore the relationship between the Cobb angle and the two loads to make their application more accurate and effective, and the results are shown in Figure 11.

As shown in Figure 11, when the Cobb angle was small, the effect of the lateral corrective load was superior to that of the axial decompressive load. However, the effect of the axial decompressive load was superior to that of the lateral corrective load in severe scoliosis. At the intersection point, where the Cobb angle was 53.3°, the corrective effects of both the loads were the same. This threshold could be used to optimize treatment strategies and minimize the required load per patient.

## 3. Discussion

This study proposed a new biomechanical model based on a self-developed 3D scoliosis decompression and correction device. The mathematical analysis of this biomechanical model revealed (1) the total axial decompressive load required to correct the scoliotic curvature and (2) the therapeutic effect of the combined action of lateral corrective and axial decompressive loads in scoliosis treatment. In addition, an analysis of the relationship between these two loads and the Cobb angle of the scoliotic curve provides robust evidence that can be used to develop and adjust treatment plans for patients with scoliosis.

The addition of lateral load during the treatment process effectively reduced the total axial load, shortened the treatment cycle, and improved the treatment effect. However, unlike surgical interventions where forces are applied directly to the spine, the transverse forces in the developed device were transmitted through the rib cage and lumbar cylinder. This might have affected the accuracy of the simulations using the developed biomechanical model, as the corrective forces in the model were applied directly and horizontally to the spine in the model without considering the indirect transmission through the rib cage and soft tissues. Currently, similar lateral load treatments have primarily been used in the form of spinal braces [23], and relatively few studies using a digitalized correction device have been conducted. Lou et al. verified the effectiveness of 3D-printed braces, but these braces are not universally applicable as different patients require personalized designs [28]. Hui et al. [29] and Rizza et al. [30] reported that the materials used in braces significantly influence treatment outcomes. The positive results of the present study further support the feasibility and effectiveness of applying axial and transverse forces in a digitalized device for conservative scoliosis treatment.

Most scoliosis braces are passive, static, or rigid and do not allow spinal mobility, potentially resulting in muscle atrophy, skin deterioration, and other spinal complications [31]. Furthermore, corrective braces cannot dynamically adjust the magnitude of lateral loads according to improvements in the Cobb angle, which might cause adverse effects. In contrast, the device model presented in the current study combines axial and lateral loads similar to those adopted in spinal braces. It does not require prolonged wearing and can adjust the magnitude and application position of the lateral load in real time according to the patient’s condition. When combined with axial loading, this approach further improved the treatment outcomes. In addition, the mathematical model allowed for individualized analysis by incorporating patient-specific parameters such as weight, age, and CT imaging data. It enabled the construction of a personalized spinal model, facilitating the development of customized plans for patients with scoliosis.

The findings of this study support the feasibility of adjusting the ratio of axial and lateral loads for different curve types to achieve desired correction outcomes. The simulation results are consistent with previous findings. Feng et al. established a 3D finite element model of the spine for patients with scoliosis and applied different correction forces to the spine model [32]. Within an appropriate range, Feng et al. found that the greater the force application, the better the correction effects. Moreover, the correction effect of the forces applied in multiple directions was better than that of forces applied in a single direction. They also proved that the relationship between torque and spinal torsion could be explored by applying moments to the scoliosis model to better study the biomechanical properties of the spine. Chen et al. studied two scoliosis curves with different Cobb angles by applying the same lateral push and axial traction forces to both the curves, and they regarded the correction effect as being generated by torque [33]. After comparison, it was found that the traction torque of the scoliosis curve with a larger Cobb angle was greater, and its lateral moment was less than that of the scoliosis curve with a smaller Cobb angle [33]. Therefore, increasing lateral forces for small-angle curves and increasing axial forces for larger-angle curves might be more efficient in enhancing correction torque. Using both torques simultaneously might also help to obtain better correction effects. The current study applied both axial and lateral loads and confirmed that for smaller Cobb angles, lateral loads were more effective, while for larger angles, axial loads had better effects.

This study proposed a preliminary biomechanical model for a novel digitalized 3D spinal decompression and correction system that primarily incorporated axial traction and lateral compression forces. Despite showing some promising results, the 2D model developed in the current study had notable limitations that required cautious interpretation and that need to be addressed in future research. More details on the study limitations and the corresponding future research efforts or directions can be found below.

Firstly, the inherently 3D nature of scoliosis was inadequately represented by the simplified 2D model, which focuses solely on transverse forces in the coronal plane and excludes some crucial components such as rotational moment, rib inclination, and sagittal alignment. This 2D simplification was a necessary compromise to establish a foundational understanding of the forces involved in spinal correction, leveraging the Timoshenko beam theory for computational feasibility and clarity. The 2D model served as a preliminary step to estimate the interaction between axial and lateral corrective forces. Future studies should extend such theoretical basis from 2D to 3D to better cope with the 3D deformity nature and 3D treatment protocol of scoliosis.

Secondly, some biomechanical parameters used in the current study presented some challenges to the model’s accuracy. For example, the bending stiffness (*EI*) values were derived from lumbar spine measurements in the sagittal plane, which may not accurately reflect the conditions in the coronal plane or the characteristics of the thoracic region. Future studies should include plane-specific and region-specific stiffness data to enhance the accuracy of developed biomechanical models.

Thirdly, the data collected from the T10-L5 segment should not be directly applied to the T2-T10 segment due to the force transmission through the ribs and the frontal orientation of the posterior joints at the thoracic level. Future studies should collect and apply data specifically from the T2-T10 segment to address this issue.

Fourthly, the biomechanical model was simplified and did not fully reflect the spine’s viscoelastic behavior. This limitation was evident in a clinical study on human subjects, which observed that the provided lateral corrective forces tended to decrease over a 28 min session of the same digitalized 3D spinal decompression and correction treatment [14]. Future studies should consider this and optimize developed biomechanical models to better reflect the spine’s natural biomechanical properties and responses.

Fifthly, the lateral forces in the model were applied directly and horizontally to the spine rather than through the rib cage (with varied obliquity at the convex side according to the patient’s condition and the severity of scoliosis) and lumbar cylinder, which is a common clinical practice in bracing treatment. The model was used to simulate the biomechanical response of an AIS patient receiving digitalized scoliosis correction, where the corrective force was applied horizontally at the thoracic level. It was unfortunate that the model in this study could not apply the corrective force according to the ribs’ inclination at the thoracic level as the rib inclination angle was not collected from the patient. Future studies could collect more data regarding the patient’s characteristics and the corrective force applied at the T2-T10 level to optimize the development of future biomechanical models. This could help improve the accuracy of the corresponding simulation results. Attention should also be paid to the influence of rib geometry on force transmission. Rib geometry could be affected by the severity of scoliosis and the level of the apex vertebra. Future studies should collect data from multiple patients with identical or comparable conditions (for example, patients with similar Cobb angle, location of curvature, and rib geometry) to facilitate the development of more applicable biomechanical models. Moreover, to enhance the model’s accuracy, future developments should also incorporate region-specific and plane-specific stiffness values, thoracic-specific biomechanical data, rotational mechanics, and sagittal plane dynamics. The lateral force directions should be adjusted according to the rib obliquity to better simulate force transmission through the rib cage. A range of anatomical models should be involved to account for the inter-individual variability in sex, age, and body habitus, which may significantly influence the biomechanical responses.

Sixthly, only a single AIS patient volunteer was recruited to validate the model’s application. Future studies could consider expanding the sample size beyond one participant and validating the model’s applicability across different scoliosis cases with similar characteristics as described above.

Seventhly, only the immediate effect of the proposed treatment method was investigated in this study. Future studies could consider incorporating a longitudinal study design, which could provide insights into the long-term effectiveness of the proposed treatment method in patients with scoliosis.

## 4. Conclusions

The scoliosis correction device described in this study uses both axial and lateral loads simultaneously to correct the curves of patients with scoliosis. This combined approach not only improves the corrective effect but also shortens the treatment period, allowing patients to better tolerate the therapy and avoid complications associated with prolonged treatment. By establishing a biomechanical model for the device, the total axial load required to correct scoliosis was identified. This facilitated the determination of an appropriate treatment duration during traction-based correction, a process informed by biomechanical analyses of spinal loading such as those in traction therapies [34]. In the current study, the total axial load gradually decreased with the application of lateral loads, which could shorten the overall treatment period. Therefore, the proposed practice of combining axial and lateral loads to correct scoliosis was found to be a safe and effective method for patients with good tolerance, and the treatment period is shorter than that when applying a single directional load. The establishment of the current biomechanical model supports the optimization and personalization of digitalized scoliosis correction devices and protocols. The findings of the current study provide a theoretical basis for individualized load planning and device adjustment based on patient-specific data.

In addition, the results of the current study indicate that when the Cobb angle of scoliosis is less than 53.3°, the corrective effect of a lateral load is better than that of an axial load, whereas when the Cobb angle exceeds 53.3°, the effect of an axial load is better than that of a lateral load. Therefore, the sequence of applying loads could be adjusted according to the severity of scoliosis. If both loads are applied simultaneously, their priority could be determined based on the patient’s Cobb angle. After determining which load plays a leading role, this load could be applied to maximize the utilization rate and effect of the device. In clinical practice, the load should be gradually increased to the recommended maximum load to obtain the optimal corrective effect, and physical examination should be performed daily to minimize complications.

## Figures and Tables

**Table 1:** Patient’s spinal coordinate data of 29 coordinate points.

**Location label**	**1**	**2**	**3**	**4**	**5**	**6**
X (mm)	0	7	14	21	28	35
Y (mm)	0	0.66	1.32	2.63	7.22	8.54
**Location label**	**7**	**8**	**9**	**10**	**11**	**12**
X (mm)	42	49	56	63	70	77
Y (mm)	9.85	11.82	14.44	19.69	23.63	24.94
**Location label**	**13**	**14**	**15**	**16**	**17**	**18**
X (mm)	84	91	98	105	112	119
Y (mm)	28.22	31.50	33.47	35.44	37.41	38.07
**Location label**	**19**	**20**	**21**	**22**	**23**	**24**
X (mm)	126	133	140	147	154	161
Y (mm)	38.72	40.04	39.38	37.41	34.13	30.85
**Location label**	**25**	**26**	**27**	**28**	**29**	
X (mm)	168	175	182	189	196	
Y (mm)	26.25	17.72	13.13	9.85	0	

**Table 2:** Detailed parameters of the volunteered scoliosis patient.

Parameters	Values
Body Weight	65 kg
Length of Scoliotic Curve (*L*)	196 mm
Bending Stiffness (*EI*)	15.1 N/m^2^
Length of Lower Part of Spine without Scoliosis (*Li*)	42 mm
Angle of Axial Load (*α*)	0°
Axial Load (*P*) (Recommended Maximum Load)	260 N
Lateral Load (*Q*) (Recommended Maximum Load)	162.5 N

## Data Availability

The original contributions presented in this study are included in the article. Further inquiries can be directed to the corresponding authors.

## References

[1] Goldberg C.J., Moore D.P., Fogarty E.E., Dowling F.E. (2008). Scoliosis: A review. Pediatr. Surg. Int..

[2] Wijngaarde C.A., Brink R.C., De Kort F.A.S., Stam M., Otto L.A.M., Asselman F.-L., Bartels B., Van Eijk R.P.A., Sombroek J., Cuppen I. (2019). Natural course of scoliosis and lifetime risk of scoliosis surgery in spinal muscular atrophy. Neurology.

[3] White A.A., Panjabi M.M. (1976). The clinical biomechanics of scoliosis. Clin. Orthop. Relat. Res..

[4] Negrini S., Aulisa A.G., Aulisa L., Circo A.B., de Mauroy J.C., Durmala J., Grivas T.B., Knott P., Kotwicki T., Maruyama T. (2012). 2011 SOSORT guidelines: Orthopaedic and Rehabilitation treatment of idiopathic scoliosis during growth. Scoliosis.

[5] Chen H., Liu C., Xu J., Maxwell A., Zhou W., Yang Y., Zhou Q., Bati A.S.R., Wan H., Wang Z. (2024). Improved charge extraction in inverted perovskite solar cells with dual-site-binding ligands. Science.

[6] You M.-J., Lu Z.-Y., Xu Q.-Y., Chen P.-B., Li B., Jiang S.-D., Jiang L.-S., Xia J., Zheng X.-F. (2024). Effectiveness of Physiotherapeutic Scoliosis-Specific Exercises on 3-Dimensional Spinal Deformities in Patients with Adolescent Idiopathic Scoliosis: A Systematic Review and Meta-analysis. Arch. Phys. Med. Rehabil..

[7] Smith T.J., Fernie G.R. (1991). Functional biomechanics of the spine. Spine.

[8] Orne D., Liu Y.K. (1971). A mathematical model of spinal response to impact. J. Biomech..

[9] Iorio J.A., Jakoi A.M., Singla A. (2016). Biomechanics of Degenerative Spinal Disorders. Asian Spine J..

[10] Nathan P., Chou S.M., Liu G. (2023). A review on different methods of scoliosis brace fabrication. Prosthet. Orthot. Int..

[11] Sun Y., Zhang Y., Ma H., Tan M., Zhang Z. (2023). Spinal Manual Therapy for Adolescent Idiopathic Scoliosis: A Systematic Review and Meta-Analysis of Randomized Controlled Trials. BioMed Res. Int..

[12] Jie Y., Li M., Dong A., Luo Y.-Y., Luo C.-L., Li J., Zheng P., Zhang X., Wong M.S., Ma C.Z. (2024). Digitalized 3D spinal decompression and correction device improved initial brace corrections and patients’ comfort among adolescents with idiopathic scoliosis: A single-centre, single-blinded randomized controlled trial. Bioengineering.

[13] Jie Y., Li M., Dong A., Luo Y.-Y., Luo C.-L., Zheng Q., Wang S., Wong M.-S., Ma C.Z.-H., Zhang M. (2025). Comparison Between a State-of-the-Art Mechanical 3D Scoliosis Correction Protocol and the Schroth Exercise on Spinal Flexibility of Patients With Adolescent Idiopathic Scoliosis: A Randomized Controlled Trial. Arch. Rehabil. Res. Clin. Transl..

[14] Luo Y., Jie Y., Luo C., Li M., Zheng Y., Wong M.S., Ma Z. Investigating a Novel Non-Surgical Management for Adolescent and Adult Scoliosis Using Digitalized 3D Correction and Ultrasound Assessment: A Preliminary Feasibility Study. Proceedings of the International Research Society of Spinal Deformities (IRSSD) Scientific Meeting 2024.

[15] Fialho J. (2018). A biomechanical model for the idiopathic scoliosis using robotic traction devices. J. Phys. Conf. Ser..

[16] Patwardhan A.G., Bunch W.H., Meade K.P., Vanderby R., Knight G.W. (1986). A biomechanical analog of curve progression and orthotic stabilization in idiopathic scoliosis. J. Biomech..

[17] Timoshenko S.P., Gere J.M., Prager W. (1962). Theory of Elastic Stability, Second Edition. J. Appl. Mech..

[18] Lee R.Y.W., Tsung B.Y.S., Tong P., Evans J. (2005). Bending stiffness of the lumbar spine subjected to posteroanterior manipulative force. J. Rehabil. Res. Dev..

[19] Hamzaoglu A., Ozturk C., Aydogan M., Tezer M., Aksu N., Bruno M.B. (2008). Posterior only pedicle screw instrumentation with intraoperative halo-femoral traction in the surgical treatment of severe scoliosis (>100 degrees). Spine.

[20] Lei L., Li W., Zhai Y. (2018). Biomechanical Analysis and Calculation of Lenke1A/B Type Scoliosis Correction. J. Med. Biomech..

[21] Wang X., Aubin C.-E.P., Labelle H., Parent S., Crandall D. (2012). Biomechanical analysis of corrective forces in spinal instrumentation for scoliosis treatment. Spine.

[22] Wang C., Xia N., Xie L., Tang Z., Huang J., Huang X. (2021). Development of a scoliosis rehabilitation robot and a preliminary study of its effectiveness in treating coronal deformity. Chin. J. Phys. Med. Rehabil..

[23] Stitzel C.J., Dovorany B., Morningstar M.W., Siddiqui A. (2014). Clinical Evaluation of the Ability of a Proprietary Scoliosis Traction Chair to De-Rotate the Spine: 6-month Results of Cobb Angle and Rotational Measurements. Clin. Pract..

[24] LaMothe J.M., Al Sayegh S., Parsons D., Ferri-De-Barros F. (2015). The Use of Intraoperative Traction in Pediatric Scoliosis Surgery: A Systematic Review. Spine Deform..

[25] Sink E.L., Karol L.A., Sanders J., Birch J.G., Johnston C.E., Herring J.A. (2001). Efficacy of perioperative halo-gravity traction in the treatment of severe scoliosis in children. J. Pediatr. Orthop..

[26] D’Astous J.L., Sanders J.O. (2007). Casting and traction treatment methods for scoliosis. Orthop. Clin. N. Am..

[27] Expert Group of Editorial Committee of Technical Guide for Prevention and Control of Abnormal Spinal Curvature in Chinese Children and Adolescents (2022). Interpretation of Technical Guidelines for Prevention and Control of Abnormal Spinal Curvature in Children and Adolescents. Chin. J. Sch. Health.

[28] Lou E., Ng K., Hill D. (2022). Immediate Outcomes and Benefits of 3D Printed Braces for the Treatment of Adolescent Idiopathic Scoliosis. Front. Rehabil. Sci..

[29] Hui C.-L., Piao J., Wong M.S., Chen Z. (2020). Study of Textile Fabric Materials used in Spinal Braces for Scoliosis. J. Med. Biol. Eng..

[30] Rizza R., Liu X.C., Thometz J., Tassone C. (2015). Comparison of biomechanical behavior between a cast material torso jacket and a polyethylene based jacket. Scoliosis.

[31] Ali A., Fontanari V., Schmölz W., Agrawal S.K. (2022). Active Soft Brace for Scoliotic Spine: A Finite Element Study to Evaluate in-Brace Correction. Robotics.

[32] Ying Y.-L., Hu Y.-X., Gao R., Yu R.-J., Gu Z., Lee L.P., Long Y.-T. (2018). Asymmetric Nanopore Electrode-Based Amplification for Electron Transfer Imaging in Live Cells. J. Am. Chem. Soc..

[33] Chen Z.-Q., Wang C.-F., Bai Y.-S., Zhu X.-D., Yang C.-Q., Xie Y., Li M. (2011). Using precisely controlled bidirectional orthopedic forces to assess flexibility in adolescent idiopathic scoliosis: Comparisons between push-traction film, supine side bending, suspension, and fulcrum bending film. Spine.

[34] Park W.M., Kim K., Kim Y.H. (2014). Biomechanical analysis of two-step traction therapy in the lumbar spine. Man. Ther..

